# Comparative Outcomes of Standard Perioperative Eye Drops, Intravitreal Triamcinolone Acetonide-Moxifloxacin, and Intracameral Dexamethasone-Moxifloxacin-Ketorolac in Cataract Surgery

**DOI:** 10.1155/2022/4857696

**Published:** 2022-07-19

**Authors:** Robin K. Kuriakose, Soungmin Cho, Saman Nassiri, Frank S. Hwang

**Affiliations:** ^1^Loma Linda University Eye Institute, Loma Linda, CA 92354, USA; ^2^Stanford University Health Medical Center, Palo Alto, CA 94304, USA

## Abstract

**Background:**

Since the advent of cataract surgery, topical eye drops have been the mainstay of postoperative prophylaxis and treatment. Due to factors such as high expenses and poor patient compliance, there has been a growing interest and acceptance of “dropless” or “less drops” alternatives. The purpose of this study is to compare the effectiveness of intravitreal triamcinolone acetonide-moxifloxacin and intracameral dexamethasone-moxifloxacin-ketorolac to a standard eye drop regimen in controlling postoperative inflammation, corneal edema, and intraocular pressure (IOP) among cataract patients.

**Methods:**

A retrospective longitudinal comparative study among 619 consecutive eyes receiving either a standard eye drop regimen, intraoperative triamcinolone acetonide-moxifloxacin, or dexamethasone-moxifloxacin-ketorolac was performed between October 2016 and December 2020. Primary endpoints at postoperative day one (POD1), week one (POW1), and month one (POM1) included corneal edema, anterior chamber inflammation (ACI), and IOP.

**Results:**

Throughout the postoperative time points, there were no significant differences in corneal edema between intravitreal triamcinolone acetonide-moxifloxacin versus the standard eye drop therapy (OR [95% CI]: 1.09 [0.82, 1.45], *P*=0.54) and intracameral dexamethasone-moxifloxacin-ketorolac versus the standard eye drop treatment (OR [95% CI]: 1.22 [0.89, 1.67], *P*=0.22). The postoperative ACI severity was lower in the dexamethasone-moxifloxacin-ketorolac group than in the triamcinolone acetonide-moxifloxacin group by 35% on postoperative day 1 (*P*=0.01). The differences at subsequent postoperative time points were not statistically significant (*P*=0.27 and *P*=1.00 for POW1 and POM1, respectively). IOP at POM1 follow-up visit was statistically significantly higher for the triamcinolone acetonide-moxifloxacin group (mean (±SD): 15.64 (4.26)) than the dexamethasone-moxifloxacin-ketorolac (mean (±SD): 14.16 (4.02)) (*P* < 0.01). There was no statistical difference in rates of CME (*P*=0.16), and there were no cases of endophthalmitis.

**Conclusions:**

Intravitreal triamcinolone acetonide-moxifloxacin and intracameral dexamethasone-moxifloxacin-ketorolac demonstrate similar levels of efficacy to a standard eye drop regimen after cataract surgery. This study reinforces them as viable alternatives to traditional postoperative drops.

## 1. Introduction

Cataract surgery remains the most common surgical procedure in the world, with over twenty million performed worldwide [1]. With the aging population, this number will continue to rise. Advances in surgical instruments, technology, and technique have made this procedure safe with successful outcomes in most cases [2].

Since the advent of cataract surgery, topical steroids, nonsteroidal anti-inflammatory drugs (NSAIDs), and antibiotics have been the mainstay of postoperative prophylaxis and treatment. Though great visual outcomes are the primary endpoint for patients and surgeons, postoperative inflammation and microbial infection remain an overarching concern as they can lead to suboptimal or even devastating outcomes, notably postoperative infectious endophthalmitis. Citing ocular surface toxicity, high expenses, unpredictable effective dose delivery, poor compliance, and administration of topical eye drops, there has been a growing interest and acceptance of “dropless” or “less drops” alternatives [3, 4].

The European Society of Cataract and Refractive Surgeons (ESCRS) Endophthalmitis Study Group published a landmark study in 2007 demonstrating that the risk of infectious postoperative endophthalmitis was reduced by nearly 5-fold (0.34% to 0.07%) through the use of perioperative intracameral cefuroxime [5]. In another study, the rate of endophthalmitis was reported to be 0% in 25,920 subjects given intracameral antibiotics compared to other delivery methods [6]. Over the past decade, several intracameral or intravitreal preparations of antibiotics compounded with steroids have been developed. The recent advent of intravitreal triamcinolone acetonide-moxifloxacin (Tri-Moxi), composed of 1.5% triamcinolone acetonide and 0.1% moxifloxacin, offers one option for endophthalmitis prophylaxis. Recent studies have demonstrated triamcinolone acetonide-moxifloxacin as an effective modality for controlling intraocular inflammation after cataract surgery compared to a standard topical eyedrop regimen [3]. Another option used by surgeons in the US is intracameral dexamethasone-moxifloxacin-ketorolac (Dex-Moxi-Ketor), which is composed of 0.1% dexamethasone, 0.05% moxifloxacin, and 0.04% ketorolac. This compound is unique in that the preparation is clear, which prevents the transient blurring patients may experience with opaque preparations such as triamcinolone acetonide-moxifloxacin.

In this study, we compared the postoperative outcomes of triamcinolone acetonide-moxifloxacin and dexamethasone-moxifloxacin-ketorolac with a standard eye drop regimen after cataract surgery. We hypothesized that corneal edema and anterior chamber inflammation (ACI) in the intravitreal triamcinolone acetonide-moxifloxacin group and the intracameral dexamethasone-moxifloxacin-ketorolac group would show equivalent results compared to a standard eye drop regimen. We further hypothesized higher postoperative intraocular pressure in the intravitreal triamcinolone acetonide-moxifloxacin group. To our knowledge, there are no comparative studies between these newer compounded drugs and a standard eye drop regimen for cataract surgery.

## 2. Methods

### 2.1. Study Participants

This retrospective longitudinal comparative study was performed at Loma Linda University Eye Institute, Loma Linda, California, USA. Institutional Review Board approval from the Department of Human Research and Compliance at Loma Linda University Health was obtained. All methods were performed in accordance with the relevant guidelines and regulations. Informed consent and HIPAA authorization were waived due to the nature of the retrospective study. The electronic medical records were reviewed of all patients who underwent cataract surgery by a single surgeon at a tertiary medical center in Southern California from October 2016 to December 2020. The study groups included the following: (1) patients who received standard postoperative eye drop medications of an antibiotic, corticosteroid, and NSAID; (2) patients who received intraoperative intravitreal triamcinolone acetonide-moxifloxacin accompanied by a topical postoperative NSAID; and (3) patients who received intraoperative intracameral dexamethasone-moxifloxacin-ketorolac accompanied by a topical postoperative NSAID. Regimen selection was based on surgeon and patient preference, and no formal criteria were used to assign a patient to one of these groups. The following types of patients were excluded from the study: those with uncontrolled intraocular pressures (IOPs), history of steroid-responsive glaucoma and advanced glaucoma, and/or a history of intraoperative complications (e.g., posterior capsular tear and vitreous loss). Patients with a known allergy to moxifloxacin were treated with the standard eye drop regimen and a nonfluoroquinolone antibiotic substitute.

### 2.2. Procedures

Phacoemulsification using clear corneal incision was done as a routine cataract surgery in all groups. Postoperatively, all groups received one of the following NSAID regimens (depending on insurance coverage) for a total of 4 weeks: diclofenac 0.1% (4 times a day); ketorolac 0.5% (4 times a day); flurbiprofen 0.03% (4 times a day); nepafenac 0.3% (once a day); bromfenac 0.07% or 0.09% (once a day). Patients in the standard eye drop group postoperatively received topical moxifloxacin 0.5% (4 times a day for one week) and prednisolone acetate 1% (4 times a day for one week followed by a weekly taper over the next 3 weeks) along with one of the above NSAID regimens. The intravitreal triamcinolone acetonide-moxifloxacin group underwent pars plana intravitreal injection of 0.05 mL of triamcinolone acetonide-moxifloxacin 3.5 mm posterior to the limbus in the superotemporal or inferotemporal quadrant after intraocular lens implantation and before ophthalmic viscosurgical device removal. Finally, the dexamethasone-moxifloxacin-ketorolac group underwent anterior chamber fluid exchange with an intracameral injection of 0.5 mL dexamethasone-moxifloxacin-ketorolac after viscosurgical device removal, followed by hydration of the surgical wounds with this compound. All patients had at least 1-month postoperative follow-up period.

### 2.3. Assessment

Baseline demographic data were obtained preoperatively including age, sex, cataract density, uncorrected and best-corrected visual acuity (UCVA and BCVA, respectively), IOP, slit lamp, and fundus examination. In addition, postoperative clinical data were obtained such as UCVA, BCVA, IOP, ACI, corneal edema, and any postoperative complications (cystoid macular edema (CME), endophthalmitis) at postoperative day 1 (POD1), postoperative week 1 (POW1), and postoperative month 1 (POM1). Anterior chamber cell reaction was graded on a scale of 0 to 4+ using the standardization of uveitis nomenclature (SUN) grading criteria and corneal edema was graded on a scale of 0 to 4+. The presence of CME was defined as clinical evidence of macular edema on fundoscopic examination confirmed by retinal thickening on optical coherence tomography (OCT).

### 2.4. Statistical Analyses

Statistical analyses were performed using Statistical Package for the Social Sciences (SPSS) version 26 (IBM Corporation, Chicago, IL, USA) and R (R Foundation for Statistical Computing, Vienna, Austria). A Shapiro–Wilk test was used to check the normal distribution of the study variables. Summary statistics were computed for continuous (mean and median) and categorical (counts and percentages) variables. The Pearson chi-square test was conducted to compare categorical variables. The chi-square goodness-of-fit test was performed to test for equal proportional distribution in different levels of a categorical variable. A *t*-test and analysis of variance (ANOVA) were used for parametric analyses. The Kruskal–Wallis *H* test and ordinal regression statistics were applied for nonparametric analyses. General estimating equations (GEE) was used to create a regression model to compare ordinal-dependent variables of our different treatment groups throughout postoperative timepoints. Somers' *d* statistics was used for nonparametric trend analysis. A *P* value less than 0.05 was considered statistically significant.

## 3. Results

This study comprised 619 consecutive eyes of 459 patients with a median age of 71 years (range 19 to 97 years). There were more triamcinolone acetonide-moxifloxacin cases (*n* = 345), compared to dexamethasone-moxifloxacin-ketorolac (*n* = 168) and standard eye drop cases (*n* = 106). Baseline characteristics were similar between groups with the exception of a slight preponderance for a higher female percentage in the dexamethasone-moxifloxacin-ketorolac group compared to others (Table 1). The BCVA improved about 2 lines on average in our study participants 1 month after surgery compared with the preoperative values (*P* < 0.001).

Table 2 shows the odds ratios (ORs) for ACI between compounded drugs and the standard eye drop treatment at POD1, POW1, and POM1. GEE statistics demonstrated no significant differences in ACI between triamcinolone acetonide-moxifloxacin and dexamethasone-moxifloxacin-ketorolac groups with the standard eye drop treatment group throughout the postoperative time points (OR [95% CI]: 1.09 [0.82, 1.46], *P*=0.55 and OR [95% CI]: 1.19 [0.86, 1.63], *P*=0.29 for triamcinolone acetonide-moxifloxacin and dexamethasone-moxifloxacin-ketorolac groups versus standard treatment group, respectively).

**Figure 1:**
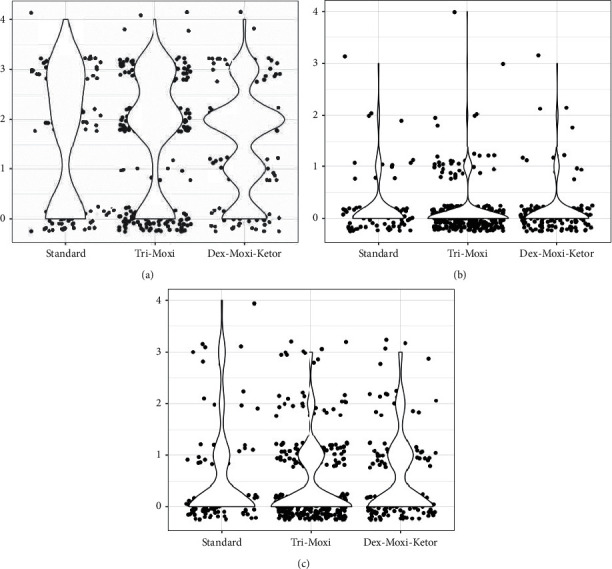
Comparison between treatment groups in anterior chamber inflammation at different postoperative timepoints.

Figure 1 demonstrates the distribution of ACI grades for different treatment groups at different postoperative time points. The trend analysis showed Somers' *d* of −0.37, −0.49, and −0.50 in decreasing postoperative intraocular inflammation severity in standard, triamcinolone acetonide-moxifloxacin, and dexamethasone-moxifloxacin-ketorolac groups, respectively, throughout the follow-up visits (all statistically significant with *P* < 0.001).

Table 3 shows the ORs for corneal edema between different compounded drugs and the standard eye drop treatment at different time points. GEE statistics demonstrated no significant differences in corneal edema between triamcinolone acetonide-moxifloxacin and dexamethasone-moxifloxacin-ketorolac groups with the standard eye drop treatment group throughout the postoperative time points (OR [95% CI]: 1.09 [0.82, 1.45], *P*=0.54, and OR [95% CI]: 1.22 [0.89, 1.67], *P*=0.22 for triamcinolone acetonide-moxifloxacin and dexamethasone-moxifloxacin-ketorolac groups versus standard treatment group, respectively). The corneal edema severity decreased on a trend of −0.42, −0.46, and −0.42 throughout the postoperative period in standard, triamcinolone acetonide-moxifloxacin, and dexamethasone-moxifloxacin-ketorolac groups, respectively (Somers' *d*, all statistically significant with *P* < 0.001).

Table 4 compares ACI and corneal edema between triamcinolone acetonide-moxifloxacin and dexamethasone-moxifloxacin-ketorolac at different time points. The postoperative anterior chamber cell reaction severity was lower in the dexamethasone-moxifloxacin-ketorolac group than in the triamcinolone acetonide-moxifloxacin group by 35% on postoperative day one (*P*=0.01). The differences at other postoperative time points were not statistically significant (*P*=0.27 and *P*=1.00 for postoperative week 1 and month 1, respectively). GEE showed no significant difference between triamcinolone acetonide-moxifloxacin versus dexamethasone-moxifloxacin-ketorolac treatment groups in either ACI or corneal edema throughout the postoperative time points (OR [95% CI]: 1.16 [0.87, 1.55], *P*=0.32, and OR [95% CI]: 1.00 [0.73, 1.36], *P*=0.98, for ACI and corneal edema, respectively).

**Figure 2:**
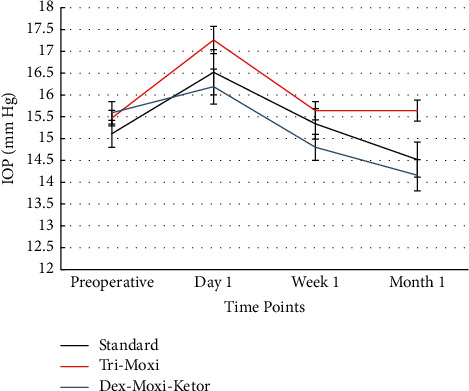
Average intraocular pressure for the treatment groups at preoperative and different postoperative timepoints. Error bars represent standard errors of the means (SEM).

Table 5 and Figure 2 compare the mean IOP between the various treatment groups at different time points postoperatively. The mean (±SD) preoperative IOPs for the standard treatment group, triamcinolone acetonide-moxifloxacin group, and the dexamethasone-moxifloxacin-ketorolac group, respectively, were as follows: 15.11 (3.10), 15.47 (3.26), and 15.59 (3.36). On average, the IOP increased by 9.3% in the standard treatment group, 11.6% in the triamcinolone acetonide-moxifloxacin group, and 3.8% in the dexamethasone-moxifloxacin-ketorolac from baseline to POD1. There was a 3.9% and 9.1% decrease from preoperative mean IOP to IOP at POM1 in the standard treatment group and the dexamethasone-moxifloxacin-ketorolac group, respectively. The triamcinolone acetonide-moxifloxacin group showed a 1.1% increase in preoperative IOP to IOP at POM1. By POM1, IOP was statistically significantly higher for the triamcinolone acetonide-moxifloxacin group than for the dexamethasone-moxifloxacin-ketorolac group (*P* < 0.01).

There were 1 (1.0%), 3 (1.2%), and 3 (4.3%) cases of CME in the standard, triamcinolone acetonide-moxifloxacin, and dexamethasone-moxifloxacin-ketorolac groups, respectively, confirmed by retinal thickening on OCT. The difference between the groups in the rate of CME was not statistically significant (*P*=0.16). There were no cases of postoperative endophthalmitis in either of the groups.

## 4. Discussion

To the best of our knowledge, this is the first comparative study assessing postoperative outcomes between a standard eye drop regimen, intravitreal triamcinolone acetonide-moxifloxacin, and intracameral dexamethasone-moxifloxacin-ketorolac for cataract surgery. During the postoperative course, inflammation and infection are the overarching concerns as they can lead to CME and endophthalmitis, respectively. Careful attention must also be paid to IOP rise and corneal edema, as these are additional factors that affect the postoperative outcomes of cataract patients.

The results of our study, based on Somers' *d* analysis, suggest that postoperative intraocular inflammation decreased at a faster rate in the dexamethasone-moxifloxacin-ketorolac group than the other groups during the first month of follow-up. GEE statistics, however, show that there are no major differences between the compounded drugs with the standard eye drops at controlling ACI throughout all postoperative time points. When comparing the two compounded drugs against each other, the postoperative ACI was significantly lower in the dexamethasone-moxifloxacin-ketorolac group than in the triamcinolone acetonide-moxifloxacin group by 35% one day postoperatively. This may be explained by the fact that the intracameral dexamethasone-moxifloxacin-ketorolac is administered directly at the target site, inherently allowing for higher drug levels in the anterior chamber early on compared to an intravitreal administration [7]. However, further prospective studies will need to be performed to validate this finding.

One of the major complications that can arise from an intracameral injection of a pharmaceutical agent is the loss of corneal endothelial cells. Our study showed no significant difference in corneal edema severity between the study groups at all postoperative time points. This suggests that either these components are nontoxic to the corneal endothelium or have little involvement in the resolution of corneal edema. These findings support prior work by Jamil et al. who found no loss in endothelial cell counts after administration of intracameral dexamethasone during cataract surgery [8]. Another study by Chan et al. found that intravitreal triamcinolone did not affect endothelial cell count in patients undergoing cataract surgery as well [9]. Finally, a study by Espiritu et al. showed that intracameral moxifloxacin 0.5% was nontoxic to the corneal endothelium, and it did not increase corneal pachymetry statistically significantly after surgery [10].

Our study demonstrated interesting findings with regards to intraocular pressures. There were no significant pressure differences between the three groups at postoperative day one and week one. All groups experienced an increase in IOP by POD1, a common trend after cataract surgery observed in prior studies [11, 12]. However, IOP at the one month postoperative follow-up was significantly higher for the triamcinolone acetonide-moxifloxacin group than in the dexamethasone-moxifloxacin-ketorolac group. One possible explanation to this could be that the intravitreal triamcinolone acetonide-moxifloxacin preparations may persist longer due to the nature of the vitreous, offering a longer therapeutic benefit but also higher susceptibility to steroid-induced pressure spike, compared to the aqueous in the anterior chamber. One study showed that given its long half-life, intraocular concentrations of triamcinolone can persist for 93 ± 28 days in nonvitrectomized eyes [13]. The presence of triamcinolone acetonide for this duration carries an increased risk of elevated intraocular pressures. Intracameral steroids such as dexamethasone have a short half-life and given its administration to the anterior chamber, a rapid turnover occurs, both in the order of hours [14]. The pharmacokinetics of these agents is not particularly well understood at this time. There was a substantial decrease in IOP from baseline to POM1 in the standard regimen group and the dexamethasone-moxifloxacin-ketorolac group, which could be accounted for by the natural trend toward lower IOP after cataract surgery compared to baseline and the fact that this intracameral medication is unlikely to cause postoperative pressure spikes as discussed earlier. The IOP lowering effect of cataract extraction with posterior chamber lens implantation has been well-studied [10, 11]. In the triamcinolone group, however, there was no significant change in IOP while comparing preoperative IOP to IOP at one month postoperatively. This suggests that perhaps there are two opposing forces at play including the presence of intravitreal triamcinolone acetonide-moxifloxacin, which can cause a pressure spike as alluded to earlier, opposing the natural IOP lowering effect of cataract surgery. A study by Ayoub et al. explored intraocular pressure outcomes in glaucoma patients and found no significant IOP elevations in patients receiving intraoperative triamcinolone acetonide-moxifloxacin compared to standard drops through six months postoperatively [15]. In our study, triamcinolone acetonide appears to negate the IOP lowering effects of cataract surgery compared to dexamethasone-moxifloxacin-ketorolac and standard regimen drops by POM1. As such, this intraoperative medication may not be suitable for patients that have elevated IOP or those with glaucoma in whom cataract surgery could be an integral part in IOP reduction.

As the duration of intraocular inflammation persists, along with risk factors such as diabetes, the rates of CME can also increase [16]. Though the rate of CME was slightly higher in the dexamethasone-moxifloxacin-ketorolac group, the difference was not statistically significant. It is important to note that this follow-up period may be inadequate as the average time for CME (Irvine–Gass syndrome) is several weeks after surgery [17]. While there were no cases of endophthalmitis in either of the study groups, cases of endophthalmitis with the use of triamcinolone acetonide-moxifloxacin have been reported [18, 19]. The aforementioned studies bolster the overall low rates of endophthalmitis with perioperative intracameral antibiotics [5, 6]. Our surgical center did experience a few cases of toxic anterior segment syndrome with the use of dexamethasone-moxifloxacin (not dexamethasone-moxifloxacin-ketorolac as evaluated in this study), likely due to a contaminated supply. This is mentioned only to highlight that compounded medications do offer some risks due to the nature of the drug preparation process.

While not specifically assessed in our study, it has been cited in other studies and by the manufacturer that triamcinolone acetonide-moxifloxacin can cause a slight visual acuity decrease and “floaters” due to the suspension nature of the preparation several days after surgery compared to the clearer preparation of dexamethasone-moxifloxacin-ketorolac. Though there were no cases of retinal detachment in our study, there is a theoretical risk with the pars plana approach for intravitreal triamcinolone acetonide-moxifloxacin compared to the intracameral dexamethasone-moxifloxacin-ketorolac. Other concerns for the routine use of these agents may include the development of antibiotic resistance [20, 21].

The findings of this study suggest noninferiority, in terms of anterior chamber inflammation, corneal edema, cystoid macular edema, and endophthalmitis. As such, the application of these intraoperative medications could be considered specifically for patients that are elderly, are noncompliant, or may have other physical or psychosocial limitations to eliminate or minimize drop administration burden. While some surgeons have used these intraoperative medications without any postoperative topical NSAIDs, making the surgery truly “dropless,” others, such as in this study, have incorporated a topical NSAID drop postoperatively to require “less drops” [13]. The cost comparison between the two injectable compound drugs explored in this study are trivial. But is cited to be significant when compared to a standard three-drop postoperative regimen [22]. Since these agents are administered intraoperatively, it is not a cost assumed by the patient. This helps to eliminate cost as a reason for patients not obtaining and administering their medications postoperatively. The cost to the patient and/or healthcare system of these prophylactic drugs can be reduced from $200–600 US dollars to $20 to $25 per case [22].

In July 2017, the American Society of Cataract and Refractive Surgery and the Academy of Ophthalmology Advisory issued a joint alert regarding possible retinal toxicity of intravitreal injection of triamcinolone acetonide-moxifloxacin from specific compounding sources. There were no such complications observed among our patients [23].

This study was particularly robust in that a single surgeon performed the surgeries, helping to eliminate some intraoperative variability that may affect corneal edema and ACI. In addition, it helps to ensure some degree of standardization among ACI grading and corneal edema during postoperative visits. Nonetheless, grading is still subjective and completed at a slit lamp. These variables could have been mitigated by the use of a corneal pachymeter and laser flare photometry. The study was large with 619 eyes included. However, the study had a small dexamethasone-moxifloxacin-ketorolac group with 168 eyes, particularly because this medication was unavailable by the manufacturer for an extended period of time. The standard regimen eye drop group was also small with only 106 eyes given the surgeon was finding good anecdotal success (including reliable use of topical drops, as well as financial and social considerations to patients) with the use of the compounded drugs. In addition, it would be beneficial to have data extending out three or more months postoperatively for a few reasons. It would enable us to capture more cases of CME as it generally takes several weeks to manifest. It would also allow us to explore how intraocular pressures may change, particularly in the intravitreal triamcinolone acetonide-moxifloxacin group. There are other intraoperative compounds not studied here, including dexamethasone-moxifloxacin or triamcinolone-moxifloxacin-vancomycin, which can be explored in the future. Finally, upcoming studies may begin to look at specific subsets of patients who may benefit more from one form of intraoperative compound compared to another.

In conclusion, our study further reinforces intraoperative administration of two specifically compounded medications with a topical NSAID as a viable alternative to traditional postoperative drops. There was a significant difference in intraocular pressure between the triamcinolone acetonide-moxifloxacin group when compared to the dexamethasone-moxifloxacin-ketorolac group at one month postoperatively. However, other outcome measures such as anterior chamber inflammation, corneal edema, and cystoid macular edema were not found to be significant one month postoperatively. Therefore, it may be prudent to avoid the use of triamcinolone acetonide-moxifloxacin in patients for whom intraocular pressures are a concern. Lastly, there appear to be no major safety concerns regarding the use of these new intraoperative compounded drugs [24].

## Figures and Tables

**Table 1:** Baseline characteristics of the study participants.

	Standard	Tri-Moxi	Dex-Moxi-Ketor	*P* value
Number (%)	106 (17.1)	345 (55.7)	168 (27.1)	<0.001^*∗*^
Age, median (range), *y*	69 (34–97)	71 (19–97)	72 (24–90)	0.15
Female, *n* (%)	53 (52.5)	182 (52.8)	112 (67.1)	<0.01^*∗*^
Right eye, *n* (%)	54 (50.9)	183 (53.2)	73 (43.5)	0.12
Cataract density, 1-2+/3-4+, %	46.2/53.8	57.6/42.4	54.5/45.5	0.33
Preoperative IOP, median (range), mmHg	15 (8–22)	15 (5–24)	16 (5–25)	0.56
Preoperative BCVA, mean (±SD), logMAR	0.51 (0.52)	0.42 (0.23)	0.44 (0.30)	0.11

^*∗*^Statistically significant.

**Table 2:** Anterior chamber inflammation comparison between compound drugs and standard eyedrop treatment at different time point.

	Day 1		Week 1		Month 1	
	OR (95% CI)^*∗*^	*P* value	OR (95% CI)^*∗*^	*P* value	OR (95% CI)^*∗*^	*P* value
Tri-Moxi	1.21 (0.81, 1.80)	0.36	1.03 (0.65, 1.64)	0.90	0.74 (0.37, 1.46)	0.38
Dex-Moxi-Ketor	0.81 (0.52, 1.26)	0.35	1.28 (0.76, 2.13)	0.35	0.74 (0.33, 1.66)	0.46

^*∗*^Reference = Standard eyedrop treatment.

**Table 3:** Corneal edema comparison between compound drugs and standard eyedrop treatment at different time point.

	Day 1		Week 1		Month 1	
	OR (95% CI)^*∗*^	*P* value	OR (95% CI)^*∗*^	*P* value	OR (95% CI)^*∗*^	*P* value
Tri-Moxi	0.95 (0.63, 1.43)	0.82	1.32 (0.81, 2.16)	0.27	1.04 (0.52, 2.05)	0.92
Dex-Moxi-Ketor	0.74 (0.47, 1.17)	0.20	1.55 (0.90, 2.66)	0.11	1.55 (0.73, 3.28)	0.26

^*∗*^Reference = Standard eyedrop treatment.

**Table 4:** Anterior chamber inflammation and corneal edema comparison between Tri-Moxi and Dex-Moxi-Ketor groups at different time points.

	Day 1		Week 1		Month 1	
	OR (95% CI)^*∗*^	*P* value	OR (95% CI)^*∗*^	*P* value	OR (95% CI)^*∗*^	*P* value
Anterior chamber inflammation	0.65 (0.46, 0.91)	0.01^†^	1.24 (0.85, 1.82)	0.27	1.00 (0.51, 1.96)	1.00
Corneal edema	0.77 (0.54, 1.09)	0.14	1.18 (0.80, 1.7)	0.41	1.50 (0.86, 2.61)	.016

^*∗*^Reference = Tri-Moxi treatment. ^†^Statistically significant.

**Table 5:** Intraocular pressure comparison between different treatment groups at different time points.

	Standard	Tri-Moxi	Dex-Moxi-Ketor	*P* value
IOP (PreOp), mean (±SD)	15.11 (3.10)	15.47 (3.26)	15.59 (3.36)	0.49
IOP (POD1), mean (±SD)	16.52 (5.26)	17.26 (5.69)	16.19 (5.17)	0.09
IOP (POW1), mean (±SD)	15.34 (3.50)	15.64 (3.77)	14.80 (3.83)	0.07
IOP (POM1), mean (±SD)	14.52 (3.84)	15.64 (4.26)	14.16 (4.02)	0.001^*∗*^

^*∗*^Statistically significant.

## Data Availability

Data are available on request through contact with the IRB at Loma Linda University or through any of the aforementioned authors.

## Abbreviations

IOP: Intraocular pressure
ACI: Anterior chamber inflammation
POD1: Postoperative day one
POW1: Postoperative week one
POM1: Postoperative month one
CME: Cystoid macular edema
UCVA: Uncorrected visual acuity
BCVA: Best-corrected visual acuity
OCT: Optical coherence tomography
SD: Standard deviation
OR: Odds ratio.
